# Anaerobic Digestion and Removal of Sulfamethoxazole, Enrofloxacin, Ciprofloxacin and Their Antibiotic Resistance Genes in a Full-Scale Biogas Plant

**DOI:** 10.3390/antibiotics10050502

**Published:** 2021-04-28

**Authors:** Andrea Visca, Anna Barra Caracciolo, Paola Grenni, Luisa Patrolecco, Jasmin Rauseo, Giulia Massini, Valentina Mazzurco Miritana, Francesca Spataro

**Affiliations:** 1 Water Research Institute, National Research Council (IRSA-CNR), 00010 Montelibretti, Italy; visca@irsa.cnr.it (A.V.); grenni@irsa.cnr.it (P.G.); mazzurco@irsa.cnr.it (V.M.M.); 2 Institute of Polar Sciences, National Research Council (ISP-CNR), 00010 Montelibretti, Italy; luisa.patrolecco@cnr.it (L.P.); jasmin.rauseo@cnr.it (J.R.); francesca.spataro@cnr.it (F.S.); 3 Department of Energy Technologies, Italian National Agency for New Technologies, Energy and Sustainable Economic Development (ENEA), 00123 Rome, Italy; giulia.massini@enea.it

**Keywords:** antibiotics, degradation, ARGs, fluoroquinolones, sulfonamides, digestate, zootechnical waste

## Abstract

Anaerobic digestion is one of the best ways to re-use animal manure and agricultural residues, through the production of combustible biogas and digestate. However, the use of antibiotics for preventing and treating animal diseases and, consequently, their residual concentrations in manure, could introduce them into anaerobic digesters. If the digestate is applied as a soil fertilizer, antibiotic residues and/or their corresponding antibiotic resistance genes (ARGs) could reach soil ecosystems. This work investigated three common soil emerging contaminants, i.e., sulfamethoxazole (SMX), ciprofloxacin (CIP), enrofloxacin (ENR), their ARGs *sul1, sul2, qnrS, qepA, aac-(6′)-Ib-cr* and the mobile genetic element *intI1*, for one year in a full scale anaerobic plant. Six samplings were performed in line with the 45-day hydraulic retention time (HRT) of the anaerobic plant, by collecting input and output samples. The overall results show both antibiotics and ARGs decreased during the anaerobic digestion process. In particular, SMX was degraded by up to 100%, ENR up to 84% and CIP up to 92%, depending on the sampling time. In a similar way, all ARGs declined significantly (up to 80%) in the digestate samples. This work shows how anaerobic digestion can be a promising practice for lowering antibiotic residues and ARGs in soil.

## 1. Introduction

Anaerobic digestion (AD) treatment is one of the best practices for reuse of animal manure, agricultural residues and the organic fraction of municipal solid waste from the perspective of energetic valorization of waste biomass [1]. AD is a process spontaneously occurring in natural ecosystems rich in organic matter and with low oxygen content, oxidized nitrate, sulfate, iron or manganese [2]. AD can occur in natural environments such as swamps, submerged soils, wet sediments, in agroecosystems like rice fields, in confined environments such as human and animal gastrointestinal tracts (large ruminant and non-ruminant herbivores, termites and woodworms) and in anthropogenic environments, such as landfills and anaerobic digesters [3]. AD comprises a sequence of metabolic reactions carried out by a complex microbial community which converts organic molecules, such as polysaccharides, lipids and proteins, into a biogas composed mainly of CH_4_ (50–75%) and CO_2_ (25–45%) [4]. Thus, one of the main advantages of the AD process when technologically implemented within anaerobic digesters is the conversion of the chemical energy in waste biomass into a biogas with a high calorific value (on average 20,000 kJ/m^3^), [5]. The combustion of biogas in cogeneration engines producing clean electricity and heat is of great interest for achieving the objectives of the new EU circular economy action plan. Furthermore, it makes it possible to reduce emission of climate-altering gases such as CH_4_ (the latter 25 times more harmful than CO_2_) [6] and environmental pollution resulting from landfills and leachate. At the end of the treatment, digestate is also obtained as a by-product of anaerobic digestion. The production of digestate is of increasing interest. It can be used as organic fertilizer [7], replacing or in combination with conventional examples [8,9] and therefore promoting organic farming and enhancing sustainable agriculture.

On the other hand, the use of antibiotics for treating and preventing animal diseases in cattle farms can be a source of soil contamination (through the feces of grazing animals or use of manure as an organic fertilizer), contributing to the environmental spreading of antibiotics and antibiotic resistance genes [10,11,12]. Because manure is commonly used as feed for anaerobic digesters, the possible presence and fate of antibiotic resistant genes (ARGs) in the AD process needs to be better investigated [13,14]. The presence of antibiotics in digesters might lead to antibiotic resistant bacteria (ARBs) and ARGs in digestate and subsequently in soil if the digestate is used as a fertilizer [15].

Among antibiotics, the sulfonamide sulfamethoxazole (SMX) and fluoroquinolones ciprofloxacin (CIP) and enrofloxacin (ENR) are widely used in human and veterinary medicine and commonly found as emerging environmental contaminants [16,17,18]. In particular, ENR is a veterinary antibiotic administered by subcutaneous injection or orally to cattle, for the treatment of infections of the respiratory and alimentary tracts. Although ENR is rapidly metabolized to CIP in animals treated, its residues (up to 1 mg/kg) can persist in soil for up to five months [19].

SMX and fluoroquinolones exhibit different behaviors in soil. SMX is relatively mobile [20] and is reported to halve in soil from 4 to 13 days from initial concentrations of 4 to 20 mg/kg [21,22,23]. On the other hand, fluoroquinolones show long-term persistence owing to their higher affinity for soil (distribution coefficients logK_Ds_: ENR from 2.7 to 3.7; CIP 2.6), [24,25]. In particular, CIP has a high affinity for soil [26] and can persist for several months [27]. The different characteristics of CIP and SMX make them good candidates for representing the entire class of antibiotics in the environment [28].

Some laboratory studies have evaluated the impact of different amounts of SMX on AD performance. A concentration of 500 mg/L completely inhibited methane production [29], while lower amounts (45-50 mg/L) led to a large accumulation of volatile fatty acids (VFA), with a consequent decrease in pH and change of the AD treatment efficiency [30]. Low antibiotic concentrations (from 1 to 10 mg/L) only partially affected the microbial community and AD process [29]. Moreover, Wang et al. [31] reported that a concentration of SMX below 40 mg/L did not significantly affect volatile fatty acid accumulation, the latter being one of the main conditions for methanogenesis inhibition.

Other studies showed that SMX can be degraded during AD, depending on specific experimental conditions and its initial concentration [22,32,33]. Recently, batch experiments showed that sulfamethoxazole not only did not inhibit the AD process, but was also degraded by a bacterial community [34].

The effect of ciprofloxacin on the activity of acetogens and methanogens in anaerobic communities was investigated by Silva et al. [35], who found acetogenic bacteria to be sensitive to ciprofloxacin concentrations above 1 mg/L, while hydrogenotrophic methanogens were not affected by any CIP concentration. Other authors [36] found that CIP caused a significant disturbance of anaerobic digestion at concentrations between 0.5 to 50 mg/L. Moreover, *Syntrophobacter* and *Methanothrix* bacteria, associated with acetoclastic methanogenesis, decreased in number. Zhi and Zhang [37] found that 10 mg/L of CIP did not affect AD, while 100 mg/L of CIP stimulated CH_4_ yield. On the other hand, concentrations of 500 mg/L generally inhibited methane yield at the initial stage of AD, even if in some cases a much higher daily CH_4_ production was observed in the late stages of the process.

However, studies of real scale anaerobic digesters showing the effects and fate of antibiotics in AD plants are not available so far [38]. In order to fill the gap and to investigate this issue, an anaerobic digester located in Central Italy was sampled and studied for one year. This full-scale plant was representative of the application of AD technology in most livestock farms. The common antibiotics SMX, ENR and CIP, together with the main ARGs responsible for SMX- (*sul1*, *sul2*), fluoroquinolone-resistance genes (*qnrS, qepA, aac-(6′)-Ib-cr*) and the mobile genetic element (MGE) *intl1*, were assessed in input and output samples. The anaerobic digester was sampled every 45 days, in line with its hydraulic retention time (HRT), to evaluate if the AD process was able to remove antibiotics and ARGs.

## 2. Results

Samplings were performed in line with the plant HRT; consequently, output samples can be considered the product of the anaerobic digestion process of a substrate input up to 45 days earlier. For this reason, each datum from an output sampling was compared with that from the corresponding previous (45 days before) input sampling.

The total microbial abundance (N. cells/g) values of input and output samples were always comparable and no significant differences were found (*t*-test non-significant). In fact, the average values (5 samplings) for input were 1.7 × 10^11^ ± 1.8 × 10^10^ cells/g and for output 7.2 × 10^11^ ± 3.8 × 10^11^ cells/g, respectively.

**Figure 1:**
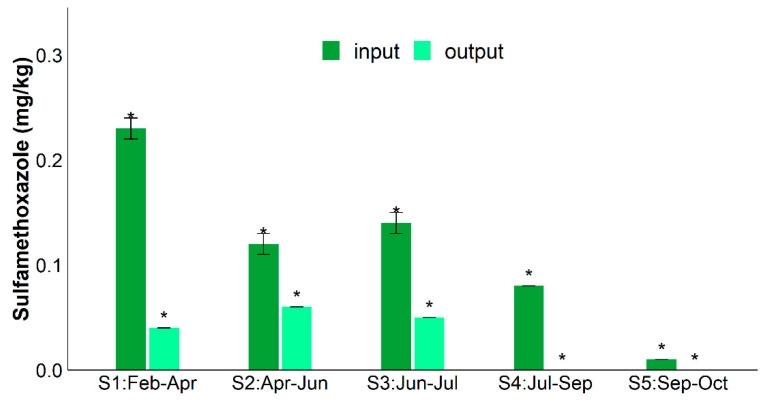
Sulfamethoxazole concentrations (mg/kg) in input and output samples collected during 2019 (S1: In_Feb_-Out_Apr_; S2: In_Apr–_Out_Jun_; S3: In_Jun–_Out_Jul_; S4: In_Jul–_Out_Sep_; S5: In_Sep–_Out_Oct_). The significant differences (*t*-test, *p* < 0.05) are marked with *.

Figure 1 shows the average SMX concentrations of input (fresh zootechnical waste) and output samples (digestate). The antibiotic concentrations, ranging from <LOD (found only in output samples) to 0.25 mg/kg, were always significantly (*p* < 0.05) higher in fresh zootechnical waste than output samples. The highest number of antibiotic residues was found in the fresh waste (input) in the winter sampling (S1).

**Figure 2:**
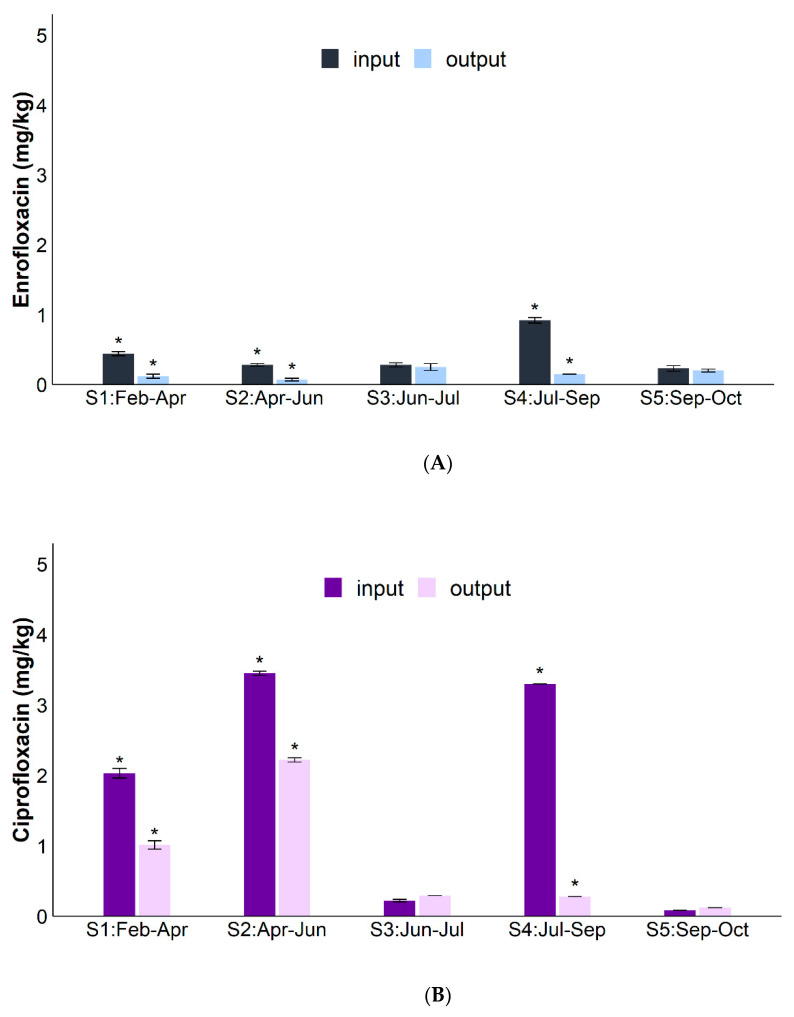
(**A**) Enrofloxacin and (**B**) Ciprofloxacin concentrations (mg/kg) in input and output samples collected during 2019 (S1: In_Feb_-Out_Apr_; S2: In_Apr–_Out_Jun_; S3: In_Jun_-Out_Jul_; S4: In_Jul_-Out_Sep_; S5: In_Sep_-Out_Oct_). The significant differences (*t*-test, *p* < 0.05) are marked with *.

Figure 2A,B show the average concentrations of ENR and CIP in input (fresh zootechnical waste) and output samples (digestate), respectively. CIP concentrations (in the range of 0.06–3.53 mg/kg) were always significantly (*p* < 0.05) higher than those of SMX (Figure 1) and of ENR (from 0.05 to 0.96 mg/kg).

As in the SMX results, CIP and ENR average concentrations were significantly (*p* < 0.05) lower in output than input samples, except for the S3 sampling.

Unlike SMX, the highest CIP and ENR concentrations were measured in summer (S4 input samples). However, in this case the removal percentages were the highest (84% ENR and 82% CIP, respectively).

Table 1 reports the removal rates calculated for SMX, CIP and ENR. The anaerobic digestion process substantially removed the antibiotics in only 45 days. The average removal efficiencies were significantly higher for SMX (78.3 ± 8.0%) than CIP (37.0 ± 25.0%) and ENR (50.3 ± 16.0%). CIP removal was highly variable, with no removal for the lowest concentrations.

**Figure 3:**
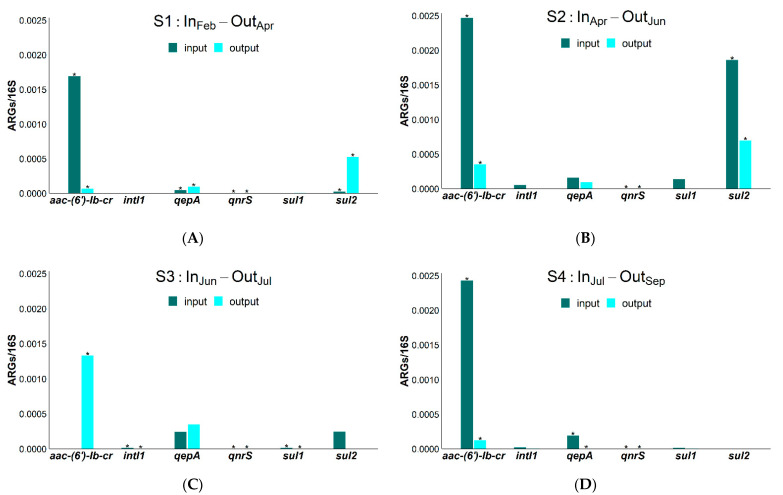

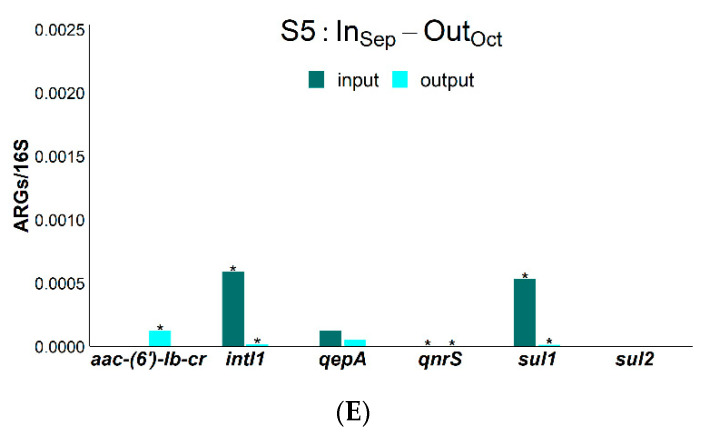
Relative gene abundances (ARGs/16S) found in (**A**) S1, (**B**) S2, (**C**) S3, (**D**) S4 and (**E**) S5 samplings (S1: In_Feb_-Out_Apr_; S2: In_Apr_-Out_Jun_; S3: In_Jun_-Out_Jul_; S4: In_Jul_-Out_Sep_; S5: In_Sep_-Out_Oct_). Significant differences (*t*-test, *p* < 0.05) are marked with *.

Figure 3A–E report the CIP and SMX genes investigated in the input (fresh waste) and output samples (digestate). All genes (*sul1, sul2, qnrS, qepA, aac-(6′)-Ib-cr, intI1*) searched for were found, even if at variable abundances, depending on the sampling and the specific gene considered. The relative abundances of *sul1* and *intI1* were generally quite low; the *sul2* gene was higher than *sul1* and *intI1*. In the case of fluoroquinolones, *aac-(6′)-Ib-cr* was the most abundant gene (from 4.8 × 10^−5^ to 1.2 × 10^−1^); in fact, *qnrS* and *qepA* were found from 0 to 3.4 × 10^−7^ gene copies and from 2.1 × 10^−6^ to 4.3 × 10^−4^ gene copies, respectively. As regards gene abundance between input and output samples, a removal percentage was found only in the cases of the *sul1, intI1* and *aac-(6′)-Ib-cr* genes (Table 1).

## 3. Discussion

The European Medicines Agency (EMA) reports that fluoroquinolones and sulfonamides in veterinary medicine are 2.2% and 9.2%, respectively, of total antibiotics sold in Europe [39]. The amounts of veterinary antimicrobial agents sold in the various countries can be normalized in terms of Population Correction Units (PCU), i.e., the animal population potentially treated with antimicrobials. In particular, in Italy, fluoroquinolones are about 1% (2.7 mg/PCU) and sulfonamides up to 12.4% (33.9 mg/PCU) of total antibiotics sold [39]. The highest initial SMX input, measured in this study during the winter season, was presumably due to its higher consumption in this period [40]. However, the results showed that the AD process was always able to reduce SMX, whatever its initial concentration (see Table 1 and Figure 1, output samples), with an average removal of 78 ± 8%. In accordance with these results, recent batch experiments report 26% and 82% of SMX biodegraded during anaerobic digestion experiments at 15 and 69 days, respectively [34]. This result confirms that SMX is a degradable compound [41] even in anaerobic conditions with half-lives higher than in aerobic soil [23]. Moreover, in AD experiments, in which the substrate was pig manure, SMX was removed with variable elimination rates from 0% to 100%, depending on specific process parameters, including the initial antibiotic concentrations [38].

Fluoroquinolones were found in input samples in higher amounts than SMX, confirming the degradability of the latter and the persistence of CIP and ENR [42]. For example, Andriamalala et al. [28] reported CIP to be a recalcitrant compound in soil after 156 days. Similarly, Albero et al. [43] found that 70% of CIP in soil and manure-amended soil persisted 90 days after soil treatment. In the present work, in only 45 days CIP (initial concentrations from 2 to 3.5 mg/kg) was significantly removed (37 ± 25%) during the anaerobic digestion process, in line with some of the results reported in a review by Gurmessa et al. [38].

Lower initial CIP concentrations (e.g., 0.24 mg/kg in the S3 sampling) did not really decrease in the AD plant studied and this result suggests that its degradation is dependent on the concentration.

CIP concentrations were always higher than ENR, confirming that the latter, although it is the main veterinary antibiotic used, is quickly metabolized inside organisms treated to ciprofloxacin [44,45,46]. Consequently, in environmental samples, CIP residues are the result of both its direct use and that of enrofloxacin [47,48]. It is therefore desirable to look for both CIP and ENR as emerging contaminants in agroecosystems. 

The high initial input of fluoroquinolones found at the summer sampling (S4: July) can be ascribed to an unexpected occurrence of some disease in the livestock, which required their administration. In fact, ENR was presumably partially converted to CIP as mentioned above and as found in other research [49]. Interestingly, a peak in the *aac-(6′)-Ib-cr* gene, which encodes for an aminoglycoside acetyltransferase that could acetylate ciprofloxacin, was also found in the same sampling, suggesting that this gene is sensitive to a fluoroquinolone presence.

In a similar way to *aac-(6′)-Ib-cr* results, *sul1* and *intI1* decreased in line with SMX concentrations. In fact, an average decrease in *sul1* (63.7 ± 24.0%), *aac-(6′)-Ib-cr* (89.8 ± 4.4%) and *intI1* (69.4 ± 22.5%) genes in output samples was observed. These removal percentages were in line with literature data [38]. 

On the other hand, antibiotic concentrations did not affect *sul2, qnrS* and *qepA* gene occurrence. For example, even if the SMX concentration decreased between all input and output samples, *sul2* abundance did not depend on sulfonamide presence. The *sul2* gene is usually found on small plasmids of the IncQ family [50], which are multi-resistant plasmids, and some authors [51] found that its presence could not be ascribed to a single antibiotic, but presumably to several co-selection phenomena [52]. In a similar way, the gene *qepA* was ubiquitous in both input and output samples. This can also be explained by its non-specificity; in fact, it encodes for an efflux pump able to decrease toxic accumulation (not only antibiotics) inside cells and is therefore very useful during an anaerobic digestion process for maintaining microbial cell integrity.

A general decrease in ARGs was also found in some laboratory experiments using cattle manure as a substrate for AD [38]. However, the reduction was quite variable, depending on the specific gene considered. For example, *sul1* decreased from 60% up to 78% in some cases [53,54], but in others increased from 7% up to 63% [54,55]. The *aac-(6′)-Ib-cr* gene always lessened [53,55], in accordance with our results. 

Ezzariai et al., (2018) [56] summarized several works on cattle manure composting, suggesting that this process was able to reduce antibiotic resistance genes, including SMX and CIP. However, SMX genes did not always decrease, as in the work of Qian et al., (2016) [57], which found an increase in *sul1* and *intI1*, of up to 43 times, due to an oxytetracycline spike performed before the composting. On the other hand, a slight *sul1* decrease (5%) was also found (from its initial abundance in cattle manure) in the work of Xu et al., (2017) [58]. Finally, Xie et al. [59] found fluoroquinolone resistance genes decreased by up 90% after composting in a large-scale reactor.

The variability of the removal efficiency of ARGs is due to several biotic and abiotic factors, which influence mixed microbial communities, including presence of bacterial populations able to degrade antibiotics. Moreover, waste origin (e.g., cattle, poultry and swine manure), influencing the microbial community structure [60] can also influence both antibiotic and gene removal [59,61]. For example, Xie et al. [59] found CIP to be degraded in manure after thermophilic composting from 107.1 µg/kg to 61.3 µg/kg (42% removal) and from 107.1 µg/kg to 24.2 µg/kg (77% removal) in mature cattle manure compost. In the same study, SMX degraded from 15.7 µg/kg to 6.4 µg/kg (60% removal) in thermophilic composting and from 15.7 µg/kg to 3.6 µg/kg (76% removal) in mature compost made from manure.

Finally, this work showed that the AD process, in a full-scale biogas plant, was able to remove not only ARGs, but also antibiotics (up to 91.59% of CIP and 100% of SMX), suggesting digestate as a suitable organic fertilizer. It is also desirable to use digestate for a subsequent composting process, which might further decrease antibiotic residues and resistance genes. Currently, composting anaerobic digestate is not a common practice, since digestate can respond to all requirements (“Fertilizing Product Regulation”, EU 2019/1009) of a safe and a suitable fertilizer for agroecosystems. Really, the potential environmental risks associated with the proliferation of antibiotics and ARG has so far been neglected in all organic fertilizers.

To our knowledge, this is one of the first works studying the potential capacity of an anaerobic digester to remove both antibiotics and ARGs and conducted at a full-scale anaerobic plant. The results of this work support the hypothesis of other authors [62] which mentioned anaerobic digestion as a potential biological process for removing antibiotics from livestock manure.

## 4. Materials and Methods

### 4.1. Sampling of Anaerobic Digestor

A biogas plant, located in a farm in central Italy was chosen for this study. It was selected because it is representative of the many plants (ca. 2000) currently operating in Italy. The plant consisted of two digesters placed in series operating in mesophilic conditions (33–35 °C). It was fed daily with zootechnical waste from the farm, where cattle for meat and milk were bred (Table 2). Each reactor had a working volume of 1300 m^3^ and every day an amount of 70 m^3^ was fed into it, and an equal quantity of digestate was emitted. Samplings were performed on the input and output of the plant, by collecting, respectively, feed and digestate. The samplings were performed in February (S1: 1 February), April (S2: 15 April), June (S3: 1 June), July (S4: 15 July) and September (S5: 1 September) in line with the average HRT (hydraulic retention time) of the digester of 45 days. In particular, input samples consisted of fresh zootechnical waste just before its use as feed for the reactor. Output samples consisted of digestate before its solid/liquid separation. 

At each sampling, at least three replicates (1 L each) of input and three replicates from output were collected. The samples were transported to the laboratory in refrigerated bags and immediately processed for microbiological and chemical analyses. Input and output samples were divided into sub-aliquots for different purposes. For ARG analysis and for antibiotic analytical determination samples were stored at −20 °C.

### 4.2. Chemicals and Reagents

Pure solvents (HPLC grade), such as methanol (MeOH), acetone (ACT), acetonitrile (ACN) and hydrochloric acid (37%, HCl) were purchased from VWR (Radnor, PA, USA). Formic acid (98–100%) for LC-MS LiChropur™, used to acidify the solvents and composing the mobile phase for the analytic determinations, was purchased from Sigma-Aldrich (Steinheim, Germany). The pH of the mobile phase was adjusted with a portable pH meter (HANNA Instruments, Woonsocket, RI USA). The Milli-Q Millipore system (Bedford, MA, USA) produced the ultrapure water (18 MΩ/cm quality).

SMX, CIP and ENR vetranal analytical standards were from Merck KGaA (Darmstadt, Germany). Deuterated SMX (SMX–d4, Clearsynth) and CIP (ciprofloxacin-d8 hydrochloride hydrate, Sigma-Aldrich, Steinheim, Germany) were used as internal standards. The mixing stock solution of SMX, CIP and ENR was prepared by dissolving 2.5 mg of each antibiotic in MeOH (50 mL) to obtain the final concentration of 50 mg/L and stored at −20 °C. Daily working standard solutions of antibiotics were obtained by dilution of the stock solution with a mixture of ultrapure water: MeOH (1:1 *v*/*v*) and stored at 4 °C.

Waters Oasis Hydrophilic–Lipophilic Balance (HLB) cartridges (6 mL, 1 g) were from Waters (Milford, MA, USA). The inert material used to fill the extraction cells was diatomaceous earth (Dionex™ ASE™ Prep DE) purchased from Thermo Scientific (Waltham, MA, USA).

### 4.3. Analytical Determination of SMX, CIP and ENR

The extraction of SMX, CIP and ENR from input and output samples was performed by Pressurized Liquid Extraction (PLE, E-916 Speed Extractor, Büchi, Italy) following the method described in Zhu et al. [63]. Briefly, about 2 g of fresh defrosted input and output samples were mixed and homogenized with a dispersant agent (diatomaceous earth) to fill the extraction cells. The extraction solvent was a mixture of MeOH/ACN (1:1, *v*/*v*) and the operative conditions were: temperature 80 °C, heating time 5 min, pressure 1500 psi, flush volume 60%, purge time 60 s, static cycle 1. The resulting PLE extracts were then cleaned-up/purified by SPE (Solid Phase Extraction) as in Göbel et al. [64] using the Oasis HLB cartridges. The evaluation of the analytical SMX, CIP and ENR concentrations was carried out by coupling high-performance liquid chromatography (HPLC, column Oven mod. LC-100 and micro Pump Series 200, Perkin Elmer, MA, USA) with a triple quadrupole mass spectrometer (MS/MS, API 3000, AB Sciex, Germany) equipped with an electrospray ionization source, as reported by Spataro et al. [65]. The chromatographic column consisted of a Gemini (150 × 4.6 mm, 5 μm RP C 18, Phenomenex, France), preceded by a guard column filled with the same stationary phase, both maintained at 25 °C. The injection volume and the flow rate were 20 µL and 0.3 mL/min, respectively. The separation of the analytes was obtained by gradient elution of the mobile phase composed of MeOH (phase A) and an aqueous formic acid solution (0.1%) (phase B). The chromatographic run was set as follows: 10% of phase A at 0 min, increase of phase A to 90% in 10 min, and finally the return to the initial condition in 15 min. The main MS/MS parameters set for SMX, CIP and ENR analysis are reported in Table S1 in the supplementary material.

The MS/MS operated in multiple reaction monitoring (MRM) mode. High purity N_2_ (>99.999%) was used as the collision and drying gas. The nebulizer and curtain gases were set at 14 units and 12 units, respectively. The source temperature was 400 °C and the ion-spray voltage was +5 KV. The HPLC-MS/MS system was controlled by the Analyst^®^ 1.6 Software (AB Sciex, Ontario, Canada). The same software was used for data acquisition.

The combination of m/z ion ratios, ion transition intensity ratios and the RT of the selected contaminants (criteria difference of 0.2 min) were used to identify the three antibiotics. Linearity was evaluated in the concentration range of 0.25–5 µg/L for all the antibiotics and the correlation coefficient (R^2^) was always higher than 0.98. Calibration standards (0.25, 0.5, 1.0, 2.5 and 5.0 µg/L) were prepared in triplicate for three validation runs performed on different days. The relative standard deviations of the concentrations tested were less than 15%. The addition of deuterated standards (sulfamethoxazole-d4 and ciprofloxacin-d8 hydrochloride hydrate) to working standard solutions was performed for the internal standard calibration, while their addition to the purified extracts allowed the evaluation of the matrix effect and checking of the correct execution of the extraction stages.

Recovery was evaluated by spiking both input and output samples with the target antibiotics at three different concentrations (50, 100 and 250 µg/kg, five replicates). The average recovery rates for SMX, ENR and CIP were 107.6 ± 8%, 72.2 ± 5% and 64.5 ± 6%, respectively.

The limits of detection (LOD) for the SMX, ENR and CIP antibiotics, calculated in accordance with IUPAC [66], were 0.5 µg/kg, 0.6 µg/kg and 0.6 µg/kg, respectively. The quantification limits were set as three times LOD values.

### 4.4. Total Microbial Abundance

Total microbial abundances (N. cells/g) of input or output samples were measured with the epifluorescence direct count method, as reported in previous works [34]. This method made it possible to quantify the overall cell abundance considering 1 g (dry weight) of each input and output sample.

### 4.5. DNA Extraction

Total DNA was extracted from input and output samples (1 g per replicate) using the DNeasy PowerSoil kit (Qiagen, Germantown Road Germantown, MD, USA) following the manufacturer’s recommendations. DNA-free water was included as a negative control during the entire workflow. The quantity and quality of the DNA extracted were assessed with spectrophotometry (Multiskan Sky Microplate Spectrophotometer, Thermo Fisher Scientific, MA, USA). After extraction, DNA was stored at −20 °C until use.

### 4.6. Quantification of ARGs and intI1 Sequences by qPCR

qPCR was performed on the CFX96 real-time PCR detection system (Bio-Rad, United States) as reported previously [34], targeting the genes *sul1, sul2, qnrS, qepA, aac-(6′)-Ib-cr, intI1* and *16S rRNA*. The primers here used are already reported by other authors [67,68,69,70,71,72,73] and are listed in Table S2. All ARG and *intI1* qPCR results were normalized per 16S rRNA gene copies (relative abundance).

### 4.7. Antibiotic and ARG Removal

Antibiotic removal and ARG loss (R) were obtained by using the following equation:

(1) $$R=\frac{\left(W_{f}-W_{a}\right)}{W_{f}}\times 100$$

where *W_f_* and *W_a_* are the antibiotic or ARG amount found in zootechnical effluents (input) and digestate (output), respectively, and *R* indicates the loss of antibiotic or ARG expressed as a percentage [74].

### 4.8. Statistical Analysis

The microbiological and chemical results are reported as average ± standard errors of triplicate analysis of triplicate samples. Paired t-tests were performed with R software to evaluate the variation between two series of numerical samples at the statistical significance level of 0.05 (*p* ≤ 0.05, confidence interval 95%).

## 5. Conclusions

The overall results suggest that the AD process favored a general decrease, not only in SMX, CIP and ENR concentrations, but also in the resistance genes related to their presence. Even if the antibiotic and ARG residues in output samples were variables, presumably owing to the overall differences operating in the digester (e.g., several antibiotics and microbial populations introduced with various organic inputs), the application of digestate seems a practice more desirable than that of manure for reducing antibiotic soil contamination.

## Figures and Tables

**Table 1:** SMX, CIP, ENR, ARGs and *intI1* gene removal percentage (R %) calculated between input and output samples. S1: In_Feb_-Out_Apr_; S2: In_Apr_-Out_Jun_; S3: In_Jun_-Out_Jul_; S4: In_Jul_-Out_Sep_; S5: In_Sep_-Out_Oct._

	R_SMX_ (%)	R_ENR_ (%)	R_CIP_ (%)	R*_sul1_* (%)	R*_aac-(6′)-Ib-cr_* (%)	R*_intI1_* (%)
S1	82.9	71.9	50.3	-	96.2	-
S2	50.3	75.8	35.6	97.2	85.8	95.8
S3	63.9	10.9	-	98.3	72.3	98.8
S4	94.3	83.9	91.6	50.6	94.9	73.9
S5	100.0	8.9	-	97.6	99.9	97.0
Average	78.3 ± 8.0%	50.3 ± 16.0%	37.0% ± 25.0%	64.0 ± 24.0%	90.0 ± 5.0%	69.0 ± 22.0%

Removal percentage with negative values are reported as “-“.

**Table 2:** Main characteristic of the farm.

Farm Type	No. of Animals	Cattle Breed	Feeding of Cattle	Manure Storage	Digestate Treatment
Dairy farm	700	Dairy Friesian	Corn shredded, triticale, soya, cotton seeds, corn flour	Open air pool	Solid/liquid separation

## Data Availability

Not Applicable.

## Supplementary Materials

The following are available online at https://www.mdpi.com/article/10.3390/antibiotics10050502/s1, Table S1: Main MS/MS parameters set for the detection of SMX, ENR and CIP. RT: retention time; ESI mode means positive (+) ionization mode; DP: declustering potential; CE: collision energy. Table S2: List of primers used for ARG quantification.
