# Supplementary material for: Anaerobic Digestion and Removal of Sulfamethoxazole, Enrofloxacin, Ciprofloxacin and Their Antibiotic Resistance Genes in a Full-Scale Biogas Plant
Source: Antibiotics (Basel). 2021 Apr 28;10(5):502. doi: 10.3390/antibiotics10050502 (PMC8146758; doi:10.3390/antibiotics10050502)
Supplement: Supplementary file 1 [file antibiotics-10-00502-s001.zip › antibiotics-1176031-supplementary.pdf]

Table S1. Main MS/MS parameters set for the detection of SMX, ENR and CIP. RT means retention time; ESI mode means positive (+) ionization mode; DP means declustering potential; CE means collision energy.

**Compound**	**RT (min)**	**ESI Mode**	**Precursor ion (m/z)**	**Product ion (m/z)**	**DP (V)**	**CE (eV)**
SMX	6.4	+	254.1	92.0<br>108.1<br>156.0	40	39.0<br>39.4<br>22.8
ENR	13.8	+	360.2	342.1<br>316.1	40	21.0<br>35.0
CIP	14.5	+	332.2	245.2<br>288.2	40	33.6<br>26.5

Table S2. List of primers used for ARGs quantification

**Primer name**	***Target gene***	***Primer sequence (5′->3′)***	***Reference***
Sul1 fw	*sul1*	*CGCACCGGAAACATCGCTGCAC*	[67]
Sul1 rv		*TGAAGTTCCGCCGCAAGGCTCG*	
Sul2 fw	*sul2*	*GCGCTCAAGGCAGATGGCATT*	[68]
Sul2 rv		*GCGTTTGATACCGGCACCCGT*	
IntI1 fw	*intI1*	*TCGTGCGTCGCCATACA*	[69]
IntI1 rv		*GCTTGTTCTACGGCCGTTTGA*	
16S fw	*16S rRNA*	*CGGTGAATACGTTCYCGG*	[70]
16S rv		*TACCTTGTTACGACTT*	
qnrS fw	*qnrS*	*GACGTGCTAACTTGCGTGAT*	[71]
qnrS rv		*TGGCATTGTTGGAAACTT*	
qepA fw	*qepA*	*GCAGGTCCAGCAGCGGGTAG*	[72]
qepA rv		*CTTCCTGCCCGAGTATCGTG*	
Aac-(6′)-Ib cr fw	*aac-(6′)-Ib-cr*	*TGCATCACAACTGGGCAAAGGCT*	[73]
Aac-(6′)-Ib cr rv		*ACACGGCTGGACCATATGGGGT*	
